# Development of a Novel Anti-CD44 Variant 5 Monoclonal Antibody C_44_Mab-3 for Multiple Applications against Pancreatic Carcinomas

**DOI:** 10.3390/antib12020031

**Published:** 2023-04-28

**Authors:** Yuma Kudo, Hiroyuki Suzuki, Tomohiro Tanaka, Mika K. Kaneko, Yukinari Kato

**Affiliations:** 1 Department of Molecular Pharmacology, Tohoku University Graduate School of Medicine, 2-1 Seiryo-machi, Aoba-ku, Sendai 980-8575, Miyagi, Japan; 2 Department of Antibody Drug Development, Tohoku University Graduate School of Medicine, 2-1 Seiryo-machi, Aoba-ku, Sendai 980-8575, Miyagi, Japan

**Keywords:** CD44, CD44 variant 5, monoclonal antibody, flow cytometry, immunohistochemistry

## Abstract

Pancreatic cancer exhibits a poor prognosis due to the lack of early diagnostic biomarkers and the resistance to conventional chemotherapy. CD44 has been known as a cancer stem cell marker and plays tumor promotion and drug resistance roles in various cancers. In particular, the splicing variants are overexpressed in many carcinomas and play essential roles in the cancer stemness, invasiveness or metastasis, and resistance to treatments. Therefore, the understanding of each CD44 variant’s (CD44v) function and distribution in carcinomas is essential for the establishment of CD44-targeting tumor therapy. In this study, we immunized mice with CD44v3–10-overexpressed Chinese hamster ovary (CHO)-K1 cells and established various anti-CD44 monoclonal antibodies (mAbs). One of the established clones (C_44_Mab-3; IgG_1_, kappa) recognized peptides of the variant-5-encoded region, indicating that C_44_Mab-3 is a specific mAb for CD44v5. Moreover, C_44_Mab-3 reacted with CHO/CD44v3–10 cells or pancreatic cancer cell lines (PK-1 and PK-8) by flow cytometry. The apparent *K*_D_ of C_44_Mab-3 for CHO/CD44v3–10 and PK-1 was 1.3 × 10^−9^ M and 2.6 × 10^−9^ M, respectively. C_44_Mab-3 could detect the exogenous CD44v3–10 and endogenous CD44v5 in Western blotting and stained the formalin-fixed paraffin-embedded pancreatic cancer cells but not normal pancreatic epithelial cells in immunohistochemistry. These results indicate that C_44_Mab-3 is useful for detecting CD44v5 in various applications and is expected to be useful for the application of pancreatic cancer diagnosis and therapy.

## 1. Introduction

Pancreatic cancer has become the third leading cause of death in men and women combined in the United States in 2023 [1]. The development of pancreatic cancer has been explained by four common oncogenic events, including *KRAS*, *CDKN2A*, *SMAD4*, and *TP53* [2,3]. However, pancreatic cancer shows a heterogeneity in drug response and clinical outcomes [4]. Therefore, detailed understanding of pancreatic cancers has been required to improve patient selection for current therapies and to develop novel therapeutic strategies. An integrated genomic analysis of pancreatic ductal adenocarcinomas (PDAC) was performed and defined four subtypes, including squamous, pancreatic progenitor, immunogenic, and aberrantly differentiated endocrine exocrine (ADEX), which correspond to the histopathological characteristics [5]. Additionally, various marker proteins have been investigated for the early diagnostic and drug responses of pancreatic cancers [6]. Studies have suggested that CD44 plays important roles in malignant progression of tumors through its cancer stemness and metastasis-promoting properties [7,8].

CD44 is a type I transmembrane glycoprotein that is expressed as a wide variety of isoforms in various types of cells. [9]. The variety of isoforms is produced by the alternative splicing of CD44 mRNA. The CD44 standard isoform (CD44s) is the smallest isoform of CD44 (85–95 kDa); it is presented on the membrane of most vertebrate cells. CD44s mRNA is assembled by the first five and the last five constant region exons [10]. The CD44 variant isoforms (CD44v) are produced by the alternative splicing of middle variant exons (v1–v10) and the standard exons of CD44s [11]. CD44v is heavily glycosylated, leading to various molecular weights (~250 kDa) owing to *N*-glycosylation and *O*-glycosylation [12]. Both CD44s and CD44v (pan-CD44) are known as hyaluronic acid (HA) receptors that mediate cellular homing, migration, adhesion, and proliferation [13].

CD44v is overexpressed in carcinomas and induce metastatic properties [14,15]. A growing body of evidence suggests that CD44v plays critical roles in the promotion of tumor invasion, metastasis, cancer-initiating properties [16], and resistance to chemo- and radiotherapy [7,17]. Reports indicated the important functions of each variant’s exon-encoded region. The v3-encoded region functions as a co-receptor for receptor tyrosine kinases [18]. Since the v3-encoded region possesses heparan sulfate moieties, it can recruit to heparin-binding epidermal growth factor-like growth factor (HB-EGF) and fibroblast growth factors (FGFs). Furthermore, the v6-encoded region forms a ternary complex with HGF and its receptor c-MET, which is essential for its activation [19]. Additionally, oxidative stress resistance is mediated by the v8–10-encoded region through binding with a cystine–glutamate transporter (xCT) subunit [20]. Therefore, establishment and characterization of mAbs that recognize each CD44v is thought to be essential for understanding each variant’s function and development of CD44-targeting tumor diagnosis and therapy. However, the function and distribution of the variant-5-encoded region in tumors has not been fully understood.

Our group established the novel anti-pan-CD44 mAbs, C_44_Mab-5 (IgG_1_, kappa) [21] and C_44_Mab-46 (IgG_1_, kappa) [22] using the Cell-Based Immunization and Screening (CBIS) method and immunization with the CD44v3–10 ectodomain, respectively. Both C_44_Mab-5 and C_44_Mab-46 have epitopes within the standard exon (1 to 5)-encoding sequences [23,24,25]. Furthermore, we showed that both C_44_Mab-5 and C_44_Mab-46 are applicable to flow cytometry and immunohistochemistry in oral [21] and esophageal squamous cell carcinomas (SCC) [22]. We have also investigated the antitumor effects of core-fucose-deficient C_44_Mab-5 in mouse xenograft models of oral SCC [26]. In this study, we developed a novel anti-CD44v5 mAb, C_44_Mab-3 (IgG_1_, kappa), by the CBIS method and evaluated its applications, including flow cytometry, Western blotting, and immunohistochemical analyses.

## 2. Materials and Methods

### 2.1. Cell Lines

Chinese hamster ovary (CHO)-K1 and mouse multiple myeloma P3X63Ag8U.1 (P3U1) cell lines were obtained from the American Type Culture Collection (ATCC, Manassas, VA, USA). The human pancreas cancer cell lines PK-1 and PK-8 were obtained from the Cell Resource Center for Biomedical Research Institute of Development, Aging and Cancer at Tohoku University. These cells were cultured in Roswell Park Memorial Institute (RPMI)-1640 medium (Nacalai Tesque, Inc., Kyoto, Japan) supplemented with 100 U/mL penicillin, 100 μg/mL streptomycin, 0.25 μg/mL amphotericin B (Nacalai Tesque, Inc.), and 10% heat-inactivated fetal bovine serum (FBS; Thermo Fisher Scientific, Inc., Waltham, MA, USA). All the cells were grown in a humidified incubator at 37 °C with 5% CO_2_.

### 2.2. Plasmid Construction and Establishment of Stable Transfectants

CD44v3–10 open reading frame was obtained from the RIKEN BRC through the National Bio-Resource Project of the MEXT, Japan. CD44s cDNA was amplified using the HotStar HiFidelity Polymerase Kit (Qiagen Inc., Hilden, Germany) and LN229 (a glioblastoma cell line) cDNA as a template. CD44v3–10 and CD44 cDNAs were subcloned into pCAG-Ble-ssPA16 vectors with a signal sequence and N-terminal PA16 tag of 16 amino acids (GLEGGVAMPGAEDDVV) [21,27,28,29,30]; this can be detected by NZ-1, which was originally developed as an anti-human podoplanin mAb [31,32,33,34,35,36,37,38,39,40,41,42,43,44,45,46]. The pCAG-Ble/PA16-CD44s and pCAG-Ble/PA16-CD44v3–10 vectors were transfected into CHO-K1 cells using a Neon transfection system (Thermo Fisher Scientific, Inc.), which offers an innovative electroporation method that utilizes a proprietary biologically compatible pipette tip chamber to generate a more uniform electric field for a significant increase in transfection efficiency and cell viability. By the limiting dilution method, CHO/CD44s and CHO/CD44v3–10 clones were finally established.

### 2.3. Hybridomas

The female BALB/c mice were purchased from CLEA Japan (Tokyo, Japan). All animal experiments were approved by the Animal Care and Use Committee of Tohoku University (Permit number: 2019NiA-001) and performed according to relevant guidelines and regulations to minimize animal suffering and distress in the laboratory. The mice were intraperitoneally immunized with CHO/CD44v3–10 (1 × 10^8^ cells) and Imject Alum (Thermo Fisher Scientific Inc.) as an adjuvant. After the three additional immunizations per week, a booster injection was performed two days before harvesting the spleen cells of immunized mice. The hybridomas were established by the fusion of splenocytes and P3U1 cells using polyethylene glycol 1500 (PEG1500; Roche Diagnostics, Indianapolis, IN, USA). RPMI-1640 supplemented with hypoxanthine, aminopterin, and thymidine (HAT; Thermo Fisher Scientific Inc.) was used for the selection of hybridomas. The supernatants, which are negative for CHO-K1 cells and positive for CHO/CD44v3–10 cells, were selected by flow cytometry using SA3800 Cell Analyzers (Sony Corp. Tokyo, Japan).

### 2.4. Enzyme-Linked Immunosorbent Assay (ELISA)

Fifty-eight synthesized peptides, covering the CD44v3–10 extracellular domain [23], were synthesized by Sigma-Aldrich Corp. (St. Louis, MO, USA). The peptides (1 µg/mL) were immobilized on Nunc Maxisorp 96-well immunoplates (Thermo Fisher Scientific Inc.). Plate washing was performed with phosphate-buffered saline (PBS) containing 0.05% (*v*/*v*) Tween 20 (PBST; Nacalai Tesque, Inc.). After blocking with 1% (*w*/*v*) bovine serum albumin (BSA) in PBST, C_44_Mab-3 (10 µg/mL) was added to each well. Then, the wells were further incubated with peroxidase-conjugated anti-mouse immunoglobulins (1:2000 dilution; Agilent Technologies Inc., Santa Clara, CA, USA). One-Step Ultra TMB (Thermo Fisher Scientific Inc.) was used for enzymatic reactions. An iMark microplate reader (Bio-Rad Laboratories, Inc., Berkeley, CA, USA) was used to mesure the optical density at 655 nm.

### 2.5. Flow Cytometry

CHO-K1, CHO/CD44v3–10, PK-1, and PK-8 were obtained using 0.25% trypsin and 1 mM ethylenediamine tetraacetic acid (EDTA; Nacalai Tesque, Inc.). The cells were incubated with C_44_Mab-3, C_44_Mab-46, or blocking buffer (control) (0.1% BSA in PBS) for 30 min at 4 °C. Then, the cells were treated with Alexa Fluor 488-conjugated secondary antibody (Cell Signaling Technology, Inc., Danvers, MA, USA) for 30 min at 4 °C. The data were analyzed using the SA3800 Cell Analyzer and SA3800 software ver. 2.05 (Sony Corp.).

### 2.6. Determination of Dissociation Constant (K_D_) via Flow Cytometry

CHO/CD44v3–10 and PK-1 cells were treated with serially diluted C_44_Mab-3 (0.01–10 µg/mL). Then, the cells were incubated with Alexa Fluor 488-conjugated secondary antibody. Fluorescence data were analyzed using BD FACSLyric and BD FACSuite software version 1.3 (BD Biosciences, Franklin Lakes, NJ, USA). The *K*_D_ was determined by the fitting binding isotherms to built-in one-site binding models of GraphPad Prism 8 (GraphPad Software, Inc., La Jolla, CA, USA).

### 2.7. Determination of K_D_ via Surface Plasmon Resonance (SPR)

Measurement of *K*_D_ between C_44_Mab-3 and the epitope peptide was performed using SPR. C_44_Mab-3 was immobilized on the sensor chip CM5 according to the manufacturer’s protocol by Cytiva (Marlborough, MA, USA). C_44_Mab-3 (10 μg/mL in acetate buffer (pH 4.0; Cytiva)) was immobilized using an amine coupling reaction. The surface of the flow cell 2 of the sensor chip CM5 was treated with 1-ethyl-3-(3-dimethylaminopropyl)-carbodiimide and N-hydroxysuccinimide (NHS), followed by the injection of C_44_Mab-3. The *K*_D_ between C_44_Mab-3 and the epitope peptide (CD44p311–330) was determined using Biacore X100 (Cytiva). A single cycle kinetics method was used to measure the binding signals. The data were analyzed by 1:1 binding kinetics to determine the association rate constant (*k*a) and dissociation rate constant (*k*d) and *K*_D_ using Biacore X100 evaluation software (Cytiva).

### 2.8. Western Blot Analysis

The total cell lysates (10 μg of protein) were separated on 5–20% polyacrylamide gels (FUJIFILM Wako Pure Chemical Corporation, Osaka, Japan). The separated proteins were transferred onto polyvinylidene difluoride (PVDF) membranes (Merck KGaA, Darmstadt, Germany). The blocking was performed with 4% skim milk (Nacalai Tesque, Inc.) in PBST. The membranes were incubated with 10 μg/mL of C_44_Mab-3, 10 μg/mL of C_44_Mab-46, 0.5 μg/mL of NZ-1, or 1 μg/mL of an anti-β-actin mAb (clone AC-15; Sigma-Aldrich Corp.) and then incubated with peroxidase-conjugated anti-mouse immunoglobulins (diluted 1:1000; Agilent Technologies, Inc.) for C_44_Mab-3, C_44_Mab-46, and anti-β-actin. Anti-rat immunoglobulins (diluted 1:1000; Agilent Technologies, Inc.) conjugated to peroxidase was used for NZ-1. The chemiluminescence signals were obtained with ImmunoStar LD (FUJIFILM Wako Pure Chemical Corporation) and detected using a Sayaca-Imager (DRC Co., Ltd., Tokyo, Japan).

### 2.9. Immunohistochemical Analysis

One formalin-fixed paraffin-embedded (FFPE) oral SCC tissue was obtained from Tokyo Medical and Dental University [47]. FFPE sections of pancreatic carcinoma tissue arrays (Catalog number: PA241c and PA484) were purchased from US Biomax Inc. (Rockville, MD, USA). Pancreas adenocarcinoma tissue microarray with adjacent normal pancreas tissue (PA241c) contains 6 cases of pancreas adenocarcinoma with matched adjacent normal pancreas tissue, with quadruple cores per case. One oral SCC tissue was autoclaved in citrate buffer (pH 6.0; Nichirei biosciences, Inc., Tokyo, Japan), and pancreatic carcinoma tissue arrays were autoclaved in EnVision FLEX Target Retrieval Solution High pH (Agilent Technologies, Inc.) for 20 min. After blocking with SuperBlock T20 (Thermo Fisher Scientific, Inc.), the sections were incubated with C_44_Mab-3 (1 μg/mL) and C_44_Mab-46 (1 μg/mL) for 1 h at room temperature. Then, the sections were incubated with the EnVision+ Kit for mouse (Agilent Technologies Inc.) for 30 min. The color was developed using 3,3′-diaminobenzidine tetrahydrochloride (DAB; Agilent Technologies Inc.). Hematoxylin (FUJIFILM Wako Pure Chemical Corporation) was used for the counterstaining. A Leica DMD108 (Leica Microsystems GmbH, Wetzlar, Germany) was used to examine the sections and obtain images.

## 3. Results

### 3.1. Development of an Anti-CD44v5 mAb, C_44_Mab-3

**Figure 1:**
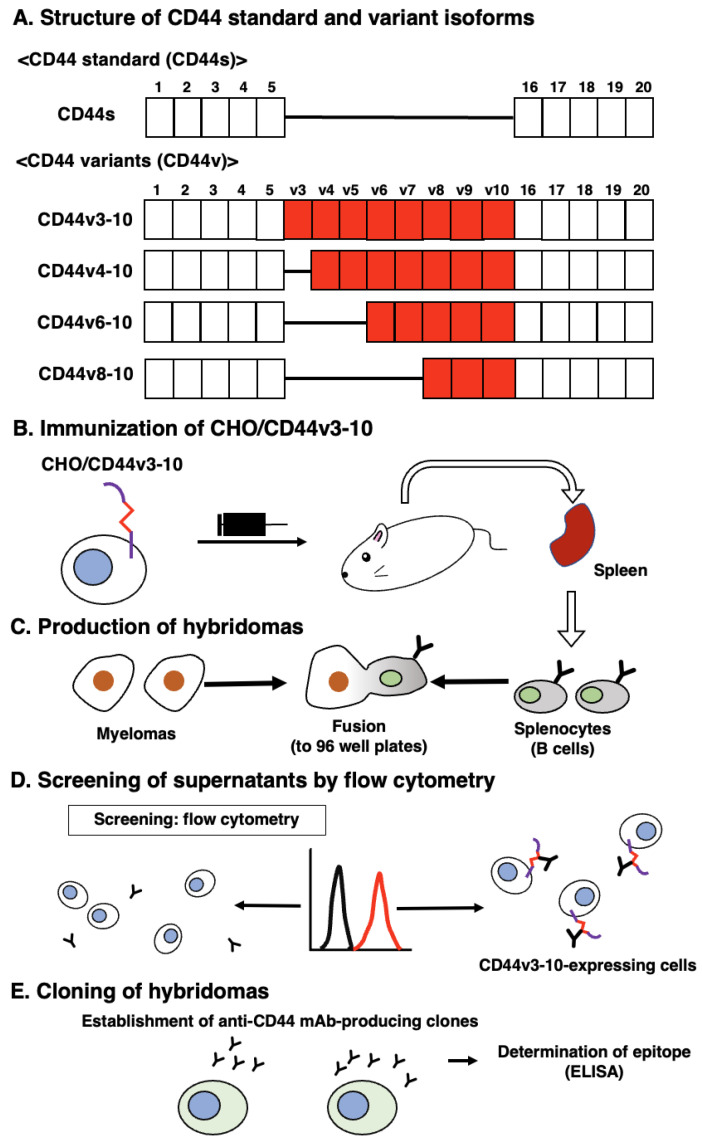
A schematic illustration of anti-human CD44 mAbs production. (**A**) Structure of CD44. CD44s mRNA is assembled by the first five (1 to 5) and the last five (16 to 20) exons and translates CD44s. The mRNAs of CD44 variants are produced by the alternative splicing of middle variant exons and translate multiple CD44v such as CD44v3–10, CD44v4–10, CD44v6–10, and CD44v8–10. (**B**) CHO/CD44v3–10 cells were intraperitoneally injected into BALB/c mice. (**C**) The splenocytes and P3U1 cells were fused and the hybridomas were produced. (**D**) The screening was conducted by flow cytometry using parental CHO-K1 and CHO/CD44v3–10 cells. (**E**) After cloning and additional screening, a clone (C_44_Mab-3 (IgG_1_, kappa)) was established. Furthermore, the binding epitope was determined by enzyme-linked immunosorbent assay (ELISA) using peptides that cover the extracellular domain of CD44v3–10.

In the CBIS method, we used a stable transfectant (CHO/CD44v3–10 cells) as an immunogen (Figure 1). Mice were immunized with CHO/CD44v3–10 cells, and hybridomas were seeded into 96-well plates. The supernatants, which are negative for CHO-K1 cells and positive for CHO/CD44v3–10 cells, were selected using flow-cytometry-based high throughput screening. By limiting dilution, anti-CD44-mAb-producing clones were finally established. Among them, C_44_Mab-3 (IgG_1_, kappa) was shown to recognize both CD44p311–330 (AYEGNWNPEAHPPLIHHEHH) and CD44p321–340 peptides (HPPLIHHEHHEEEETPHSTS), which correspond to the variant-5-encoded sequence (Table 1 and Supplementary Figure S1). In contrast, C_44_Mab-3 did not recognize other CD44v3–10 extracellular regions. These results indicated that C_44_Mab-3 specifically recognizes the CD44 variant-5-encoded sequence.

### 3.2. Flow Cytometric Analysis of C_44_Mab-3 to CD44-Expressing Cells

**Figure 2:**
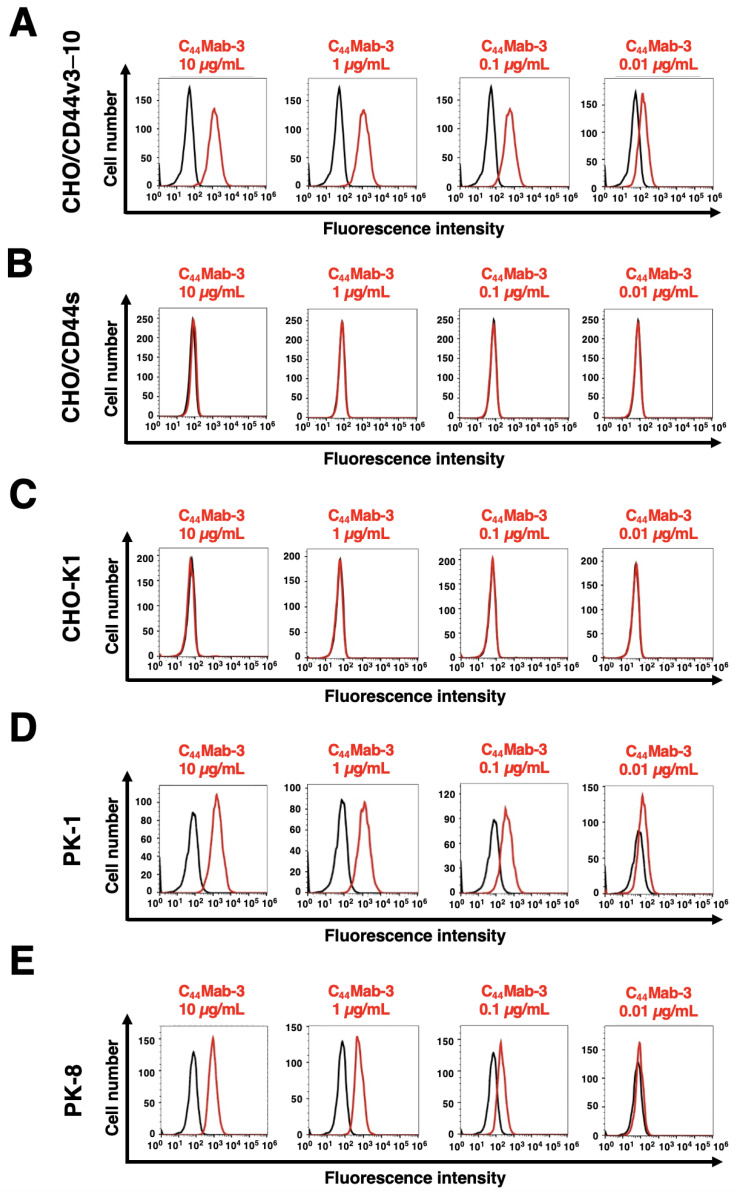
Flow cytometry using C_44_Mab-3 against CD44-expressing cells. CHO/CD44v3–10 (**A**), CHO/CD44s (**B**), CHO-K1 (**C**), PK-1 (**D**), and PK-8 (**E**) cells were treated with 0.01–10 µg/mL of C_44_Mab-3, followed by treatment with Alexa Fluor 488-conjugated anti-mouse IgG (Red line). The black line represents the negative control (blocking buffer).

We next investigated the reactivity of C_44_Mab-3 against CHO/CD44v3–10 and CHO/CD44s cells by flow cytometry. C_44_Mab-3 recognized CHO/CD44v3–10 cells in a dose-dependent manner (Figure 2A) but do not recognize either CHO/CD44s (Figure 2B) or CHO-K1 (Figure 2C) cells. An anti-pan-CD44 mAb, C_44_Mab-46 [22], recognized CHO/CD44s cells (Supplementary Figure S2). Furthermore, C_44_Mab-3 also recognized pancreatic cancer cell lines, such as PK-1 (Figure 2D) and PK-8 (Figure 2E), in a dose-dependent manner.

### 3.3. Determination of the Binding Affinity of C_44_Mab-3 by Flow Cytometry to CD44-Expressing Cells and SPR with the Epitope Peptide

**Figure 3:**
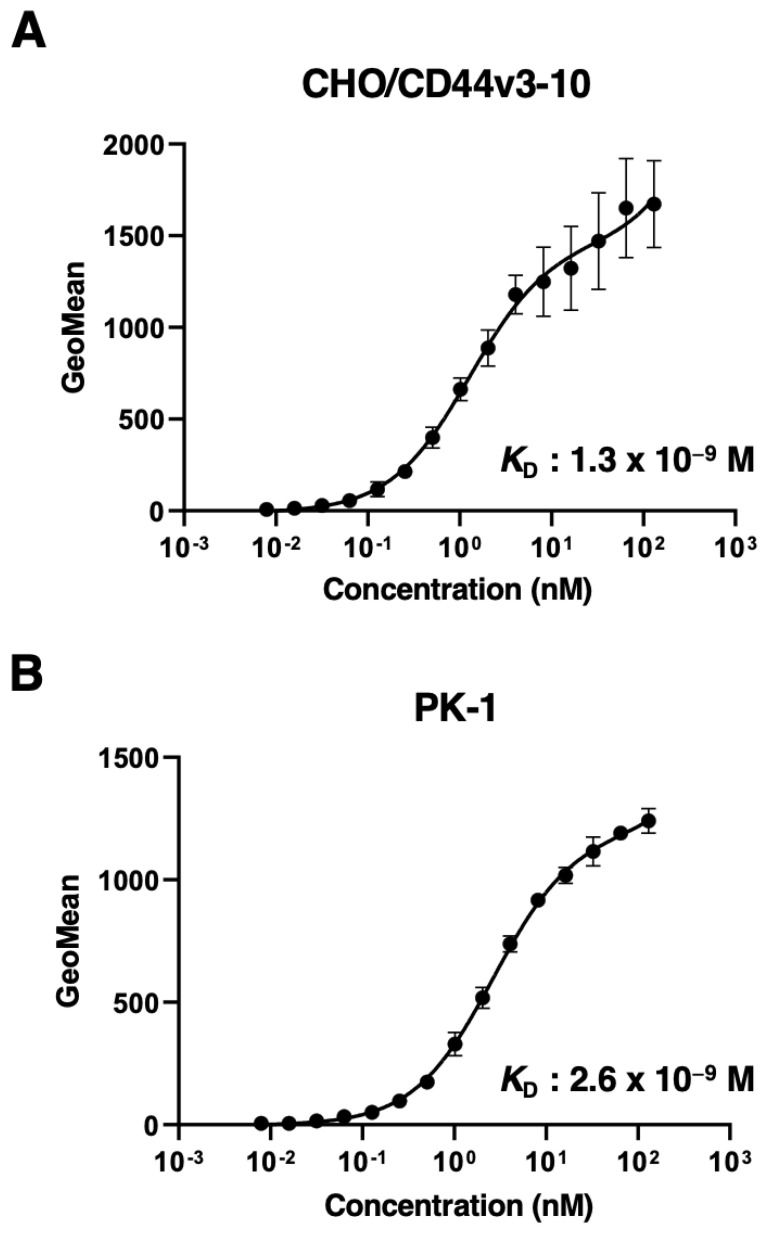
The binding affinity of C_44_Mab-3 to CD44-expressing cells. CHO/CD44v3–10 (**A**) and PK-1 (**B**) cells were suspended in 100 µL of serially diluted C_44_Mab-3 at the indicated concentrations. Then, cells were treated with Alexa Fluor 488-conjugated secondary antibody. Fluorescence data were collected and the apparent dissociation constant (*K*_D_) was calculated using GraphPad PRISM 8. Error bars represent means ± SDs.

Next, we determined the binding affinity of C_44_Mab-3 to CHO/CD44v3–10 and PK-1 using flow cytometry. As shown in Figure 3, the *K*_D_ of CHO/CD44v3–10 and PK-1 was 1.3 × 10^−9^ M and 2.6 × 10^−9^ M, respectively, indicating that C_44_Mab-3 possesses high affinity for CD44v3–10 and endogenous CD44v5-expressing cells.

We also measured the *K*_D_ of C_44_Mab-3 with the epitope peptide (CD44p311–330) using Biacore X100. The binding kinetics and measured values are summarized in Supplementary Figure S3. The *K*_D_ of CD44p311–330 was 5.5 × 10^−6^ M.

### 3.4. Western Blot Analysis

**Figure 4:**
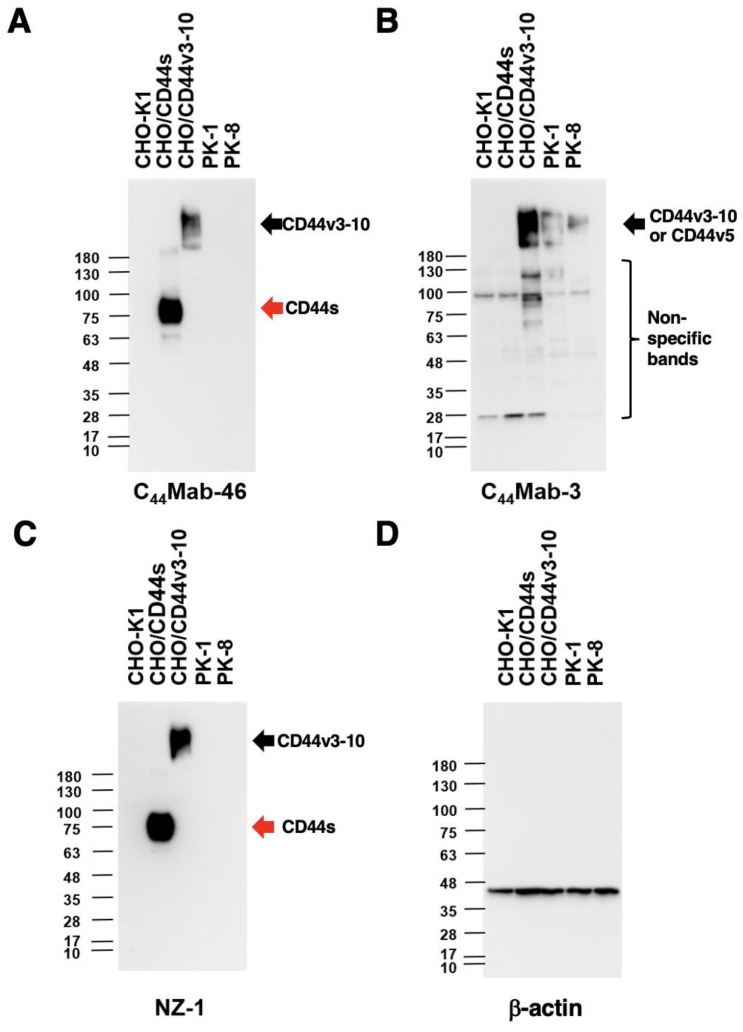
Western blot analysis using C_44_Mab-3. The cell lysates of CHO-K1, CHO/CD44s, CHO/CD44v3–10, PK-1, and PK-8 (10 µg) were electrophoresed and transferred onto polyvinylidene fluoride (PVDF) membranes. The membranes were incubated with 10 µg/mL of C_44_Mab-46 (**A**), 10 µg/mL of C_44_Mab-3 (**B**), 0.5 µg/mL of an anti-PA16 tag mAb (NZ-1) (**C**), and 1 µg/mL of an anti-β-actin mAb (**D**). Then, the membranes were incubated with anti-mouse immunoglobulins conjugated with peroxidase for C_44_Mab-46, C_44_Mab-3, and anti-β-actin. Anti-rat immunoglobulins conjugated with peroxidase were used for NZ-1. The red arrows indicate CD44s (~75 kDa). The black arrows indicate CD44v3–10 or CD44v5 (>180 kDa).

We next performed Western blot analysis to investigate the sensitivity of C_44_Mab-3. Total cell lysates from CHO-K1, CHO/CD44s, CHO/CD44v3–10, PK-1, and PK-8 were analyzed. As shown in Figure 4A, an anti-pan-CD44 mAb, C_44_Mab-46, recognized the lysates from both CHO/CD44s (~75 kDa) and CHO/CD44v3–10 (>180 kDa). C_44_Mab-3 detected CD44v3–10 as bands of more than 180-kDa. Furthermore, C_44_Mab-3 detected endogenous CD44v5-containing CD44v in PK-1 and PK-8 cells. However, C_44_Mab-3 did not detect any bands from lysates of CHO-K1 and CHO/CD44s cells (Figure 4B). An anti-PA16 tag mAb (NZ-1) recognized the lysates from both CHO/CD44s (~75 kDa) and CHO/CD44v3–10 (>180 kDa) (Figure 4C). These results indicated that C_44_Mab-3 specifically detects exogenous CD44v3–10 and endogenous CD44v5-containing CD44v.

### 3.5. Immunohistochemical Analysis Using C_44_Mab-3 against Tumor Tissues

**Figure 5:**
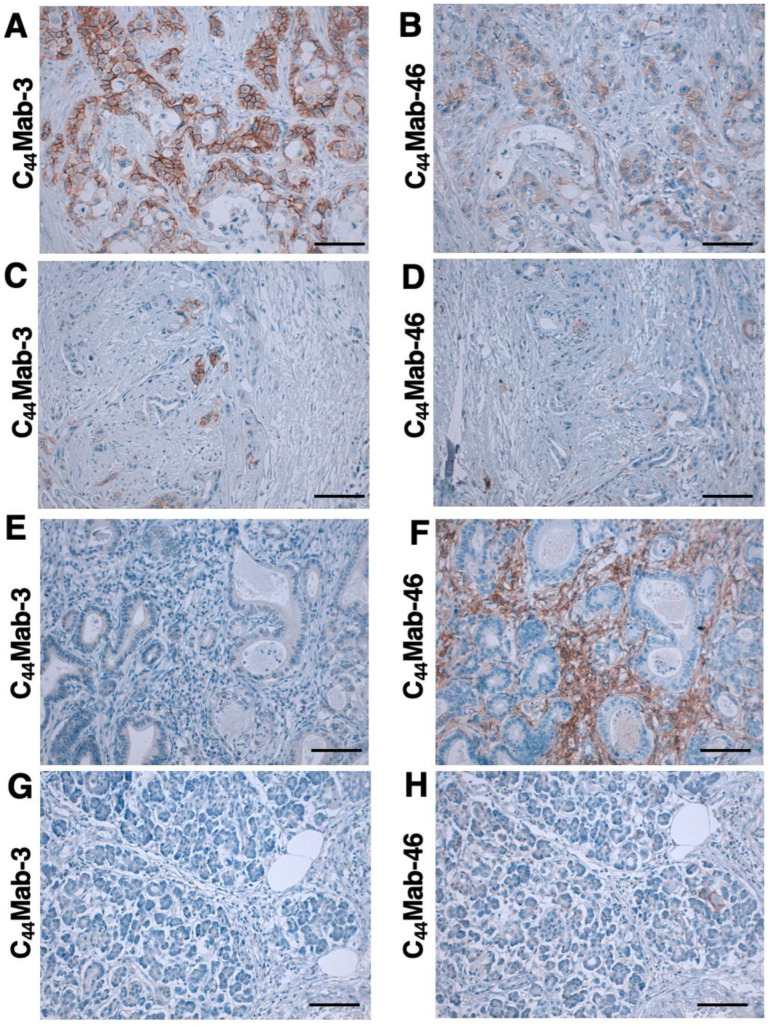
Immunohistochemical analysis using C_44_Mab-3 and C_44_Mab-46 against pancreatic adenocarcinomas and normal pancreatic tissues. After antigen retrieval, serial sections of pancreatic carcinoma tissue arrays (Catalog number: PA484) were incubated with 1 µg/mL of C_44_Mab-3 or C_44_Mab-46, followed by treatment with the Envision+ kit. The color was developed using 3,3′-diaminobenzidine tetrahydrochloride (DAB), and the sections were counterstained with hematoxylin. Scale bar = 100 µm. (**A**–**F**) pancreatic adenocarcinomas; (**G**,**H**) normal pancreas tissues.

We next examined whether C_44_Mab-3 could be used for immunohistochemical analyses using FFPE sections. We first examined the reactivity of C_44_Mab-3 and C_44_Mab-46 in an oral SCC tissue. As shown in Supplementary Figure S4, C_44_Mab-3 exhibited a clear membranous staining and could clearly distinguish tumor cells from stromal tissues. In contrast, C_44_Mab-46 stained both. We then investigated the reactivity of C_44_Mab-3 and C_44_Mab-46 in pancreatic carcinoma tissue arrays. Although we performed the antigen retrieval using citrate buffer (pH 6.0) for pancreatic carcinoma tissue arrays in the same way as with an oral SCC tissue, weak staining was observed. Therefore, we next used EnVision FLEX Target Retrieval Solution High pH for the antigen retrieval procedure; C_44_Mab-3 showed clear membranous staining in pancreatic carcinoma cells with a relatively larger cytoplasm (Figure 5A). C_44_Mab-46 also stained the same type of pancreatic carcinoma cells (Figure 5B). The staining intensity of C_44_Mab-3 was much stronger than that of C_44_Mab-46 (Figure 5A,B). Furthermore, diffusely spread tumor cells in the stroma were stained by both C_44_Mab-3 and C_44_Mab-46 (Figure 5C,D). In contrast, both C_44_Mab-3 and C_44_Mab-46 did not stain the typical ductal structure of PDAC (Figure 5E,F). In addition, stromal staining using C_44_Mab-46 was observed in several tissues (Figure 5F). Importantly, normal pancreatic epithelial cells were not stained by C_44_Mab-3 (Figure 5G). A similar staining pattern was also observed in another tissue array (Supplementary Figure S5). We summarized the data of immunohistochemical analyses in Table 2; C_44_Mab-3 stained 8 out of 20 cases (40%) (PA484, Figure 5) and 2 out of 6 cases (33%) (PA241c, Supplementary Figure S5) of pancreatic carcinomas. These results indicated that C_44_Mab-3 could be useful for immunohistochemical analysis of FFPE tumor sections and could recognize a specific type of pancreatic carcinoma.

## 4. Discussion

PDAC is the most common type of pancreatic cancer and has extremely poor prognosis, with a 5-year survival rate of approximately 10% [48]. Advances in therapy have only achieved incremental improvements in overall outcome but can provide notable benefits for undefined subgroups of patients. PDACs are heterogenous neoplasms with various histology [4] and heterogenous molecular landscapes [5]. Therefore, the identification of early diagnostic markers and therapeutic targets in each group has been desired. In this study, we developed C_44_Mab-3 using the CBIS method (Figure 1) and determined its epitope as variant-5-encoded region of CD44 (Table 1). Then, we showed the usefulness of C_44_Mab-3 for multiple applications, including flow cytometry (Figure 2 and Figure 3), Western blotting (Figure 4), and immunohistochemistry of PDAC (Figure 5).

An anti-CD44v5 mAb (clone VFF-8) was previously developed and is mainly used for the immunohistochemical analyses of tumors [49]. The epitope of VFF-8 was determined as IHHEHHEEEETPHSTST in the v5-encoded region by ELISA [50]. As shown in Table 1, C_44_Mab-3 recognized both CD44p311–330 and CD44p321–340 peptides, which commonly possess the HPPLIHHEHH sequence. The epitope of C_44_Mab-3 partially shares that of VFF-8. Further investigation of the detailed epitope mapping is required. In addition, CD44 is known to be heavily glycosylated [12], and the glycosylation pattern is thought to depend on the host cells. Since the epitope of C_44_Mab-3 does not contain serine or threonine, the recognition of C_44_Mab-3 is thought to be independent of the glycosylation.

Immunohistochemistry using VFF-8 and conventional RT-PCR analyses were performed against PDAC [49]. VFF-8 recognized PDAC but not normal pancreas cells. Furthermore, the RT-PCR analysis revealed that the exon v5 appeared in the chain containing at least v4–10 in 80% of PDACs and the cell lines tested. The authors discussed that one of the major differences between normal and PDAC was the linkage of CD44v5 to the CD44v6-containing chain [49]. Our immunohistochemical analysis also support this finding (Figure 5A,C,G). Furthermore, we found that C_44_Mab-3 could detect atypical types of PDAC, including metaplastic and diffusely invaded tumor cells (Figure 5A,C). In contrast, C_44_Mab-3 did not stain a typical ductal structure of PDAC (Figure 5E) and normal pancreatic epithelial cells (Figure 5G). In addition to conventional PDAC, the World Health Organization has classified nine histological subtypes of PDAC, which further highlight the morphologic heterogeneity of PDAC [4]. It is worthwhile to investigate whether CD44v5 is expressed in a specific subtype of PDAC in a future study.

Large-scale genomic analyses of PDACs defined four subtypes: (1) squamous; (2) pancreatic progenitor; (3) immunogenic; and (4) ADEX, which correlate with histopathological characteristics [5]. Among them, the squamous subtype is characterized as being enriched for *TP53* and *KDM6A* mutations and having upregulation of the ∆Np63 transcriptional network, hypermethylation of pancreatic endodermal determinant genes, and a poor prognosis [5]. ∆Np63 is known as a marker of basal cells of stratified epithelium and SCC [51]; it is also reported to regulate HA metabolism and signaling [52]. Specifically, ΔNp63 directly regulates the expression of CD44 through p63-binding sites that are located in the promoter region and in the first intron of CD44 gene [52]. Therefore, CD44 transcription could be upregulated in ΔNp63-positive PDAC. However, the mechanism of the variant 5 inclusion during alternative splicing remains to be determined.

Clinical trials of anti-pan-CD44 and variant-specific CD44 mAbs have been conducted [53]. An anti-pan-CD44 mAb, RG7356, exhibited an acceptable safety profile in patients with advanced solid tumors expressing CD44. However, the study was terminated due to no evidence of a clinical and pharmacodynamic dose-response relationship with RG7356 [54]. A clinical trial of a humanized anti-CD44v6 mAb bivatuzumab−mertansine drug conjugate was conducted. However, it failed due to severe skin toxicities [55,56]. The efficient accumulation of mertansine was most likely responsible for the high toxicity [55,56]. Although CD44v5 is not detected in normal pancreatic epithelium by C_44_Mab-3 (this study) and VFF-8 [49], CD44v5 could be detected in normal lung, skin, gastric, and bladder epithelium by VFF-8 [50]. For the development of the therapeutic use of C_44_Mab-3, further investigations are required to reduce the toxicity to the above tissues.

We previously converted a mouse IgG_1_ subclass of mAbs into IgG_2a_ mAb and produced defucosylated mAbs using fucosyltransferase-8-deficient CHO-K1 cells. The defucosylated IgG_2a_ mAbs showed potent antibody-dependent cellular cytotoxicity in vitro and suppressed tumor xenograft growth [26,57,58,59,60,61,62,63]. Therefore, the production of a class-switched and defucosylated version of C_44_Mab-3 is required to evaluate the antitumor activity in vivo.

## Figures and Tables

**Table 1:** Determination of the binding epitope of C_44_Mab-3 by ELISA.

Peptide	Coding Exon *	Sequence	C_44_Mab-3
CD44p21–40	2	QIDLNITCRFAGVFHVEKNG	−
CD44p31–50	2	AGVFHVEKNGRYSISRTEAA	−
CD44p41–60	2	RYSISRTEAADLCKAFNSTL	−
CD44p51–70	2	DLCKAFNSTLPTMAQMEKAL	−
CD44p61–80	2/3	PTMAQMEKALSIGFETCRYG	−
CD44p71–90	2/3	SIGFETCRYGFIEGHVVIPR	−
CD44p81–100	3	FIEGHVVIPRIHPNSICAAN	−
CD44p91–110	3	IHPNSICAANNTGVYILTSN	−
CD44p101–120	3	NTGVYILTSNTSQYDTYCFN	−
CD44p111–130	3/4	TSQYDTYCFNASAPPEEDCT	−
CD44p121–140	3/4	ASAPPEEDCTSVTDLPNAFD	−
CD44p131–150	4/5	SVTDLPNAFDGPITITIVNR	−
CD44p141–160	4/5	GPITITIVNRDGTRYVQKGE	−
CD44p151–170	5	DGTRYVQKGEYRTNPEDIYP	−
CD44p161–180	5	YRTNPEDIYPSNPTDDDVSS	−
CD44p171–190	5	SNPTDDDVSSGSSSERSSTS	−
CD44p181–200	5	GSSSERSSTSGGYIFYTFST	−
CD44p191–210	5	GGYIFYTFSTVHPIPDEDSP	−
CD44p201–220	5	VHPIPDEDSPWITDSTDRIP	−
CD44p211–230	5/v3	WITDSTDRIPATSTSSNTIS	−
CD44p221–240	5/v3	ATSTSSNTISAGWEPNEENE	−
CD44p231–250	v3	AGWEPNEENEDERDRHLSFS	−
CD44p241–260	v3	DERDRHLSFSGSGIDDDEDF	−
CD44p251–270	v3/v4	GSGIDDDEDFISSTISTTPR	−
CD44p261–280	v3/v4	ISSTISTTPRAFDHTKQNQD	−
CD44p271–290	v4	AFDHTKQNQDWTQWNPSHSN	−
CD44p281–300	v4	WTQWNPSHSNPEVLLQTTTR	−
CD44p291–310	v4/v5	PEVLLQTTTRMTDVDRNGTT	−
CD44p301–320	v4/v5	MTDVDRNGTTAYEGNWNPEA	−
CD44p311–330	v5	AYEGNWNPEAHPPLIHHEHH	+
CD44p321–340	v5	HPPLIHHEHHEEEETPHSTS	+
CD44p331–350	v5/v6	EEEETPHSTSTIQATPSSTT	−
CD44p341–360	v5/v6	TIQATPSSTTEETATQKEQW	−
CD44p351–370	v6	EETATQKEQWFGNRWHEGYR	−
CD44p361–380	v6	FGNRWHEGYRQTPREDSHST	−
CD44p371–390	v6/v7	QTPREDSHSTTGTAAASAHT	−
CD44p381–400	v6/v7	TGTAAASAHTSHPMQGRTTP	−
CD44p391–410	v7	SHPMQGRTTPSPEDSSWTDF	−
CD44p401–420	v7	SPEDSSWTDFFNPISHPMGR	−
CD44p411–430	v7/v8	FNPISHPMGRGHQAGRRMDM	−
CD44p421–440	v7/v8	GHQAGRRMDMDSSHSTTLQP	−
CD44p431–450	v8	DSSHSTTLQPTANPNTGLVE	−
CD44p441–460	v8	TANPNTGLVEDLDRTGPLSM	−
CD44p451–470	v8/v9	DLDRTGPLSMTTQQSNSQSF	−
CD44p461–480	v8/v9	TTQQSNSQSFSTSHEGLEED	−
CD44p471–490	v9	STSHEGLEEDKDHPTTSTLT	−
CD44p481–500	v9/v10	KDHPTTSTLTSSNRNDVTGG	−
CD44p491–510	v9/v10	SSNRNDVTGGRRDPNHSEGS	−
CD44p501–520	v10	RRDPNHSEGSTTLLEGYTSH	−
CD44p511–530	v10	TTLLEGYTSHYPHTKESRTF	−
CD44p521–540	v10	YPHTKESRTFIPVTSAKTGS	−
CD44p531–550	v10	IPVTSAKTGSFGVTAVTVGD	−
CD44p541–560	v10	FGVTAVTVGDSNSNVNRSLS	−
CD44p551–570	v10/16	SNSNVNRSLSGDQDTFHPSG	−
CD44p561–580	v10/16	GDQDTFHPSGGSHTTHGSES	−
CD44p571–590	16/17	GSHTTHGSESDGHSHGSQEG	−
CD44p581–600	16/17	DGHSHGSQEGGANTTSGPIR	−
CD44p591–606	17	GANTTSGPIRTPQIPEAAAA	−

+, OD655 ≥ 0.3; −, OD655 < 0.1. * The CD44 exon-encoded regions are illustrated in Figure 1.

**Table 2:** Immunohistochemical analysis using C_44_Mab-3 against pancreatic carcinoma tissue arrays.

Tissue Array	Age	Sex	Organ	Pathology Diagnosis	TNM	Grade	Stage	Type	C_44_Mab-3
PA241c	66	F	Pancreas	Adenocarcinoma	T2N0M0	1	I	malignant	+
	66	F	Pancreas	Adjacent normal pancreas tissue					–
	54	F	Pancreas	Adenocarcinoma	T3N0M0	2	II	malignant	–
	54	F	Pancreas	Adjacent normal pancreas tissue					–
	44	M	Pancreas	Adenocarcinoma	T3N0M0	2	II	malignant	–
	44	M	Pancreas	Adjacent normal pancreas tissue					–
	59	M	Pancreas	Adenocarcinoma	T2N0M0	3	I	malignant	–
	59	M	Pancreas	Adjacent normal pancreas tissue					–
	63	F	Pancreas	Adenocarcinoma	T2N0M0	3	I	malignant	+
	63	F	Pancreas	Adjacent normal pancreas tissue					–
	53	F	Pancreas	Adenocarcinoma	T3N0M0	3	II	malignant	–
	53	F	Pancreas	Adjacent normal pancreas tissue					–
PA484	35	M	Pancreas	Normal pancreas tissue	-	-	-	normal	–
	38	F	Pancreas	Normal pancreas tissue	-	-	-	normal	–
	38	M	Pancreas	Normal pancreas tissue	-	-	-	normal	–
	60	M	Pancreas	Adenocarcinoma	T3N0M0	2	II	malignant	–
	68	F	Pancreas	Adenocarcinoma	T2N0M0	2	I	malignant	+
	54	F	Pancreas	Adenocarcinoma	T3N0M0	2	II	malignant	–
	42	F	Pancreas	Adenocarcinoma	T3N0M0	2	II	malignant	–
	65	M	Pancreas	Adenocarcinoma	T3N0M0	2	II	malignant	–
	75	F	Pancreas	Adenocarcinoma	T3N0M1	2	IV	malignant	–
	57	M	Pancreas	Adenocarcinoma	T3N0M0	3	II	malignant	+
	44	M	Pancreas	Adenocarcinoma	T3N0M0	3	II	malignant	–
	47	M	Pancreas	Adenocarcinoma	T3N0M0	-	II	malignant	–
	41	M	Pancreas	Adenocarcinoma	T4N1M0	2	III	malignant	–
	64	F	Pancreas	Adenocarcinoma	T3N0M0	2	II	malignant	–
	58	F	Pancreas	Adenocarcinoma	T3N0M0	3	II	malignant	–
	47	F	Pancreas	Adenocarcinoma	T3N1M0	3	III	malignant	+
	78	M	Pancreas	Adenocarcinoma	T2N0M0	3	I	malignant	+
	49	M	Pancreas	Adenocarcinoma	T3N0M0	2	II	malignant	+
	53	F	Pancreas	Adenocarcinoma	T3N0M0	3	II	malignant	+
	60	M	Pancreas	Adenocarcinoma	T2N0M0	3	I	malignant	+
	57	F	Pancreas	Adenocarcinoma	T2N0M0	3	I	malignant	–
	61	M	Pancreas	Mucinous adenocarcinoma	T3N0M1	2	IV	malignant	–
	69	M	Pancreas	Undifferentiated carcinoma	T2N0M0	-	I	malignant	+

+, OD655 ≥ 0.3; −, OD655 < 0.1.

## Data Availability

The data presented in this study are available in the article and supplementary material.

## Supplementary Materials

The following supporting information can be downloaded at: https://www.mdpi.com/article/10.3390/antib12020031/s1, Figure S1, Determination of the binding epitope of C_44_Mab-3 by ELISA. Figure S2, Recognition of CHO/CD44s and CHO/CD44v3–10 by C_44_Mab-46 using flow cytometry. Figure S3, Measurement of dissociation constants (*K*_D_) between C_44_Mab-3 and the epitope peptide using SPR. Figure S4, Immunohistochemical analysis using C_44_Mab-3 and C_44_Mab-46 against oral squamous cell carcinoma tissue. Figure S5, Immunohistochemical analysis using C_44_Mab-3 and C_44_Mab-46 against pancreatic adenocarcinomas and normal pancreatic tissues.
