# Supplementary material for: Development of a Novel Anti-CD44 Variant 5 Monoclonal Antibody C44Mab-3 for Multiple Applications against Pancreatic Carcinomas
Source: Antibodies (Basel). 2023 Apr 28;12(2):31. doi: 10.3390/antib12020031 (PMC10204397; doi:10.3390/antib12020031)
Supplement: Supplementary file 1 [file antibodies-12-00031-s001.zip › antibodies-2217383-supplementary.pdf]

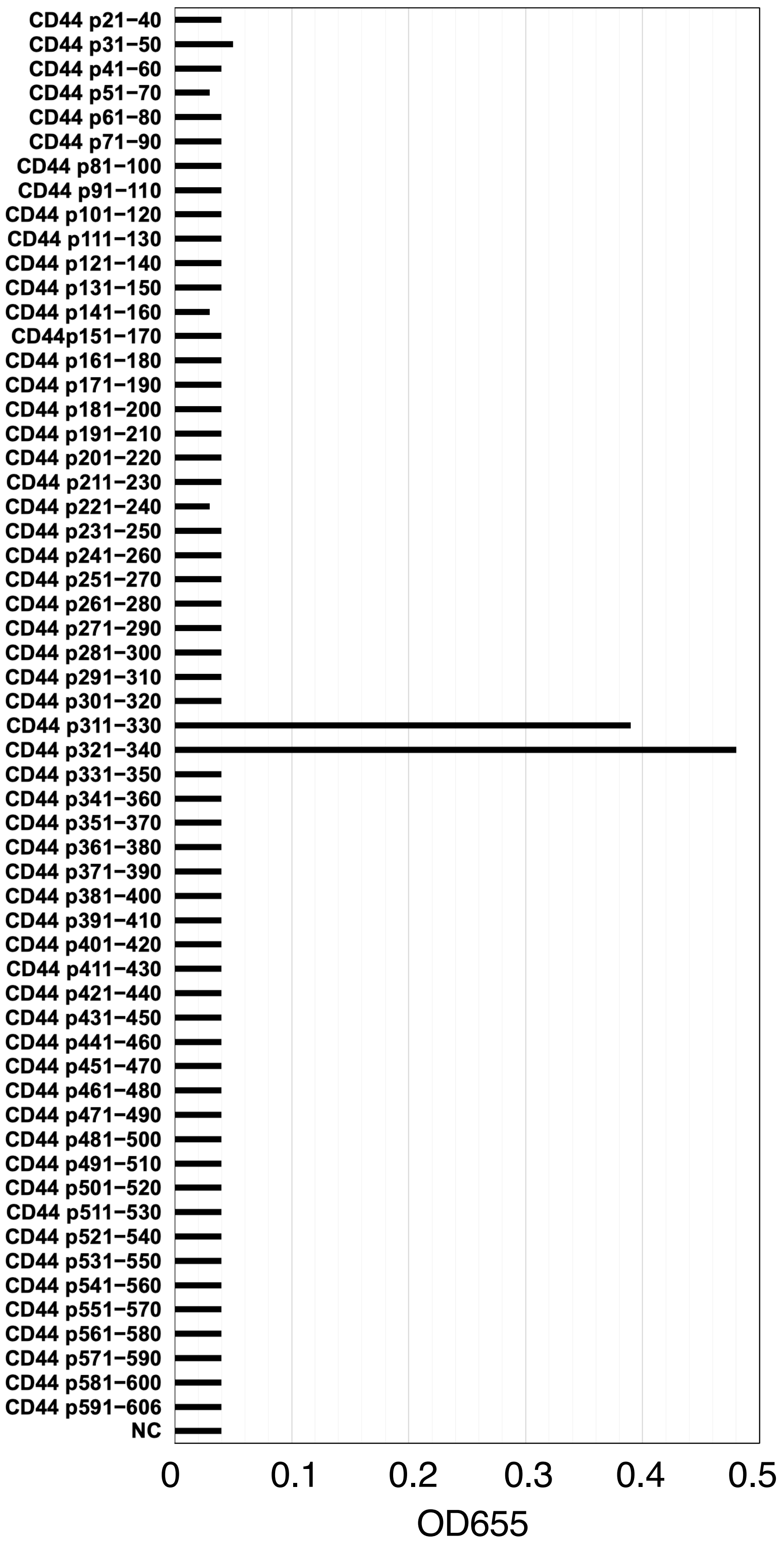

Supplementary Figure S1 Determination of C$_{44}$Mab-3 epitope by ELISA. Fifty-eight synthesized peptides, which cover the CD44v3–10 extracellular domain, were immobilized on immunoplates. The plates were incubated with C$_{44}$Mab-3, followed by incubation with peroxidase-conjugated anti-mouse immunoglobulins. Optical density was measured at 655 nm. ELISA, enzymelinked immunosorbent assay. NC, negative control (solvent; DMSO in PBS).

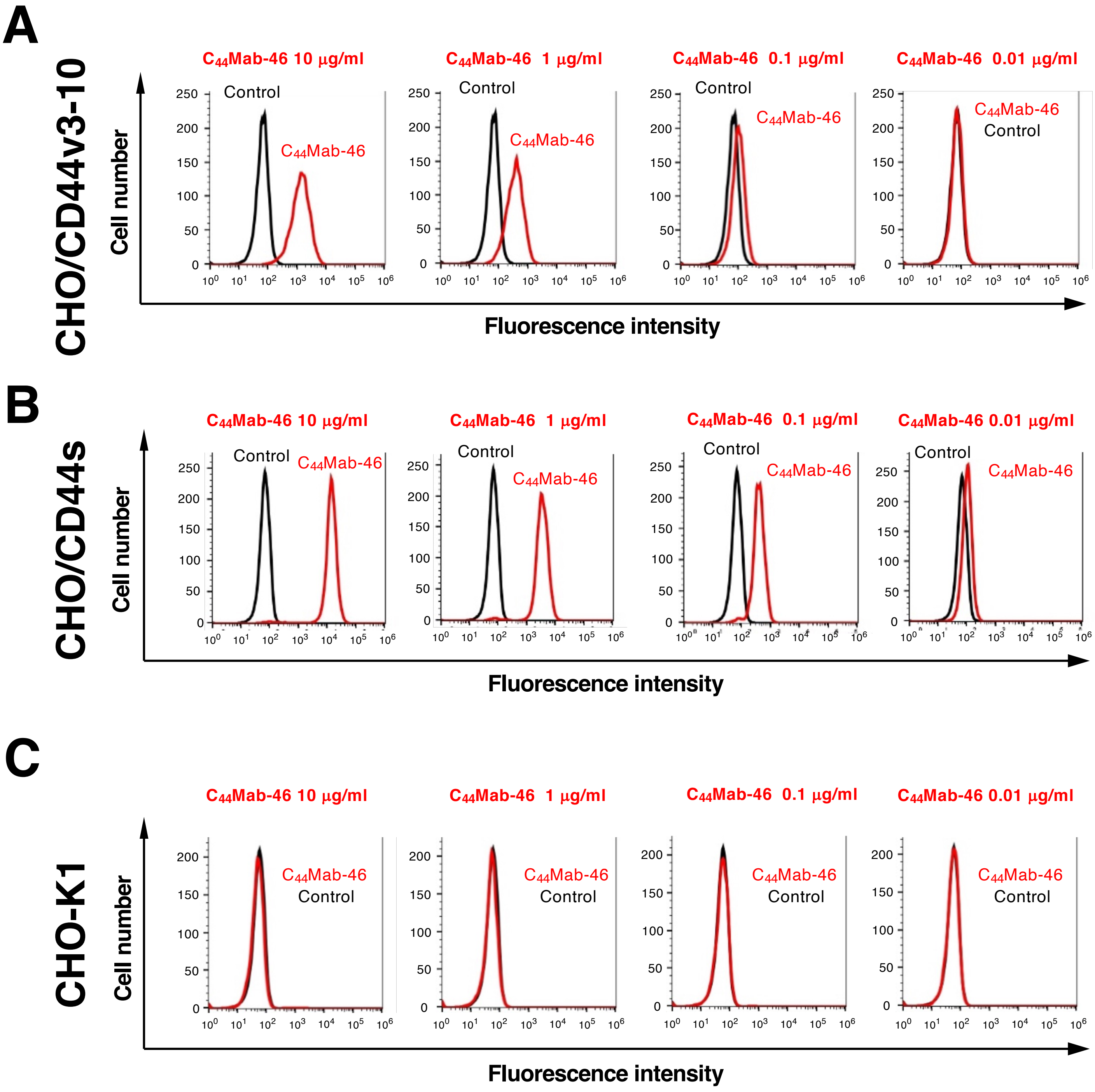

Supplementary Figure S2 Recognition of CHO/CD44s and CHO/CD44v3-10 by C_{44}Mab-46 using flow cytometry. CHO/CD44v3-10 (A), CHO/CD44s (B), and CHO-K1 (C) were treated with 0.01-10 μg/mL of C_{44}Mab-46, followed by treatment with Alexa Fluor 488-conjugated anti-mouse IgG (Red line). The black line represents the negative control (blocking buffer).

CD44p311-330

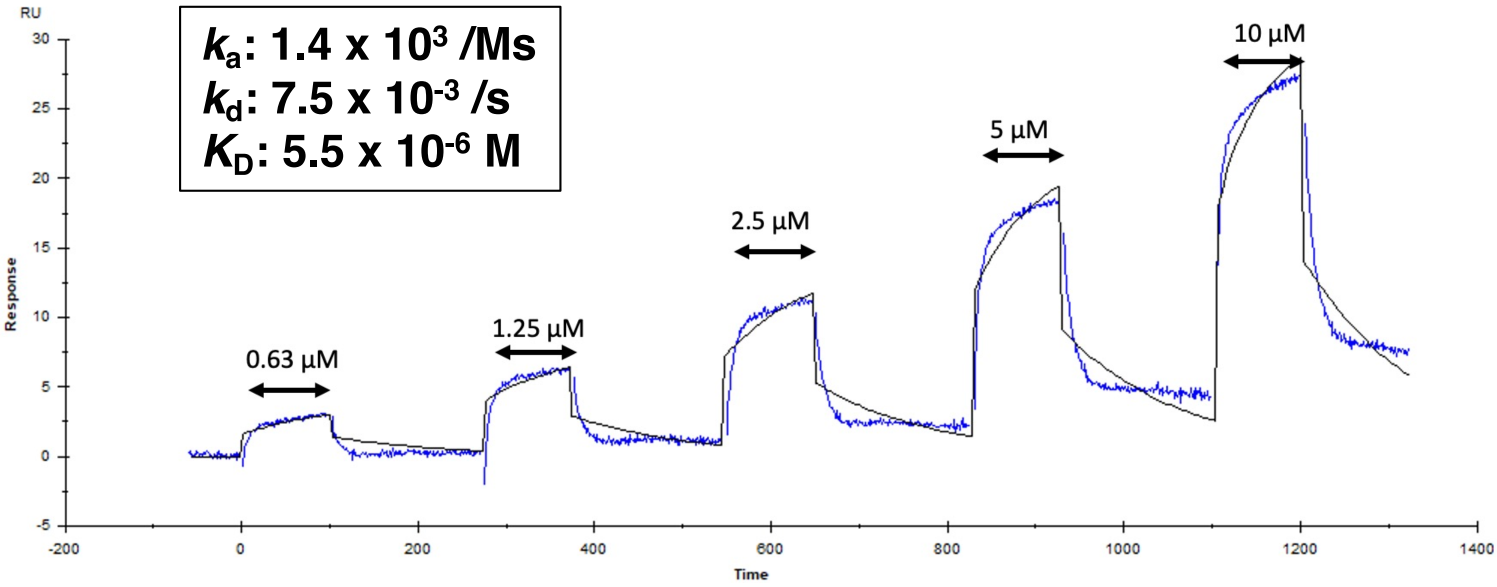

Supplementary Figure S3 Measurement of dissociation constants (K_D) between C_{44}Mab-3 and the epitope peptide using SPR.

The binding kinetics and measured values of C_{44}Mab-3 with CD44p311-330 peptide. SPR, surface plasmon resonance.

C_{44}Mab-3

C_{44}Mab-46

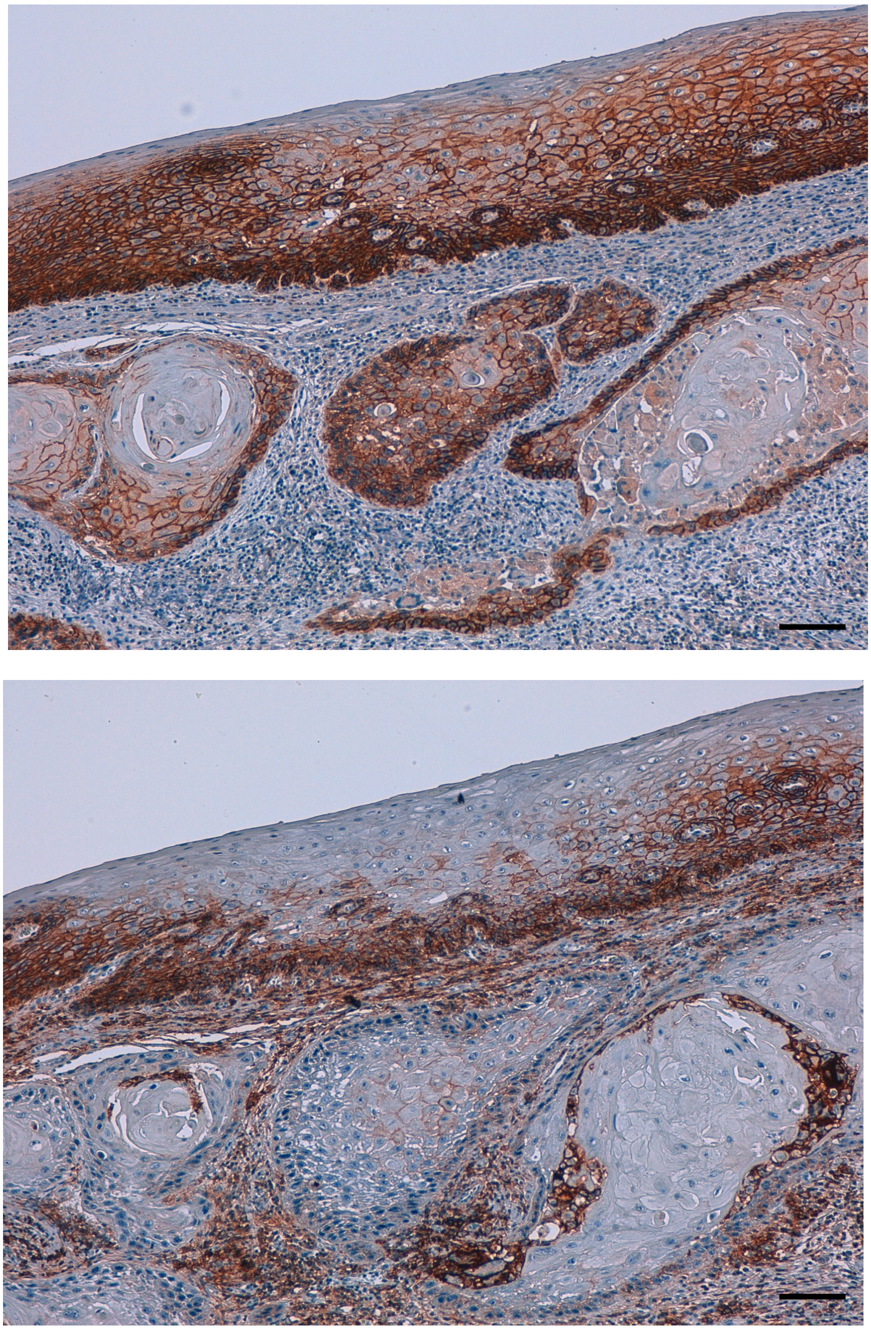

Supplementary Figure S4 Immunohistochemical analysis using C_{44}Mab-3 and C_{44}Mab-46 against oral squamous cell carcinoma tissue. After antigen retrieval, the sections were incubated with 5 μg/mL of C_{44}Mab-3 (A) and 1 μg/mL of C_{44}Mab-46 (B), followed by treatment with the Envision+ kit. The color was developed using 3,3′-diaminobenzidine tetrahydrochloride (DAB), and the sections were counterstained with hematoxylin. Scale bar = 100 μm.

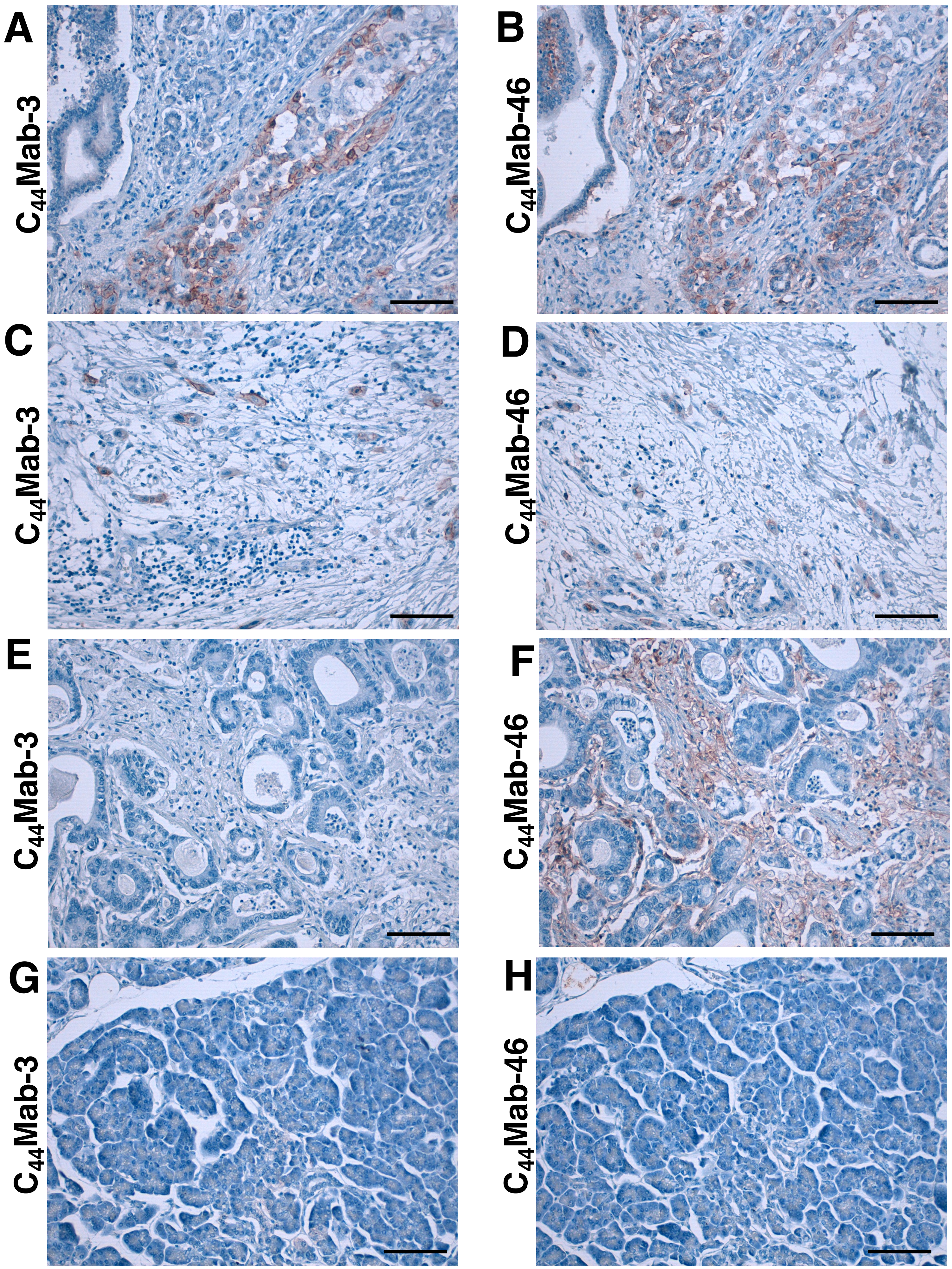
A
C44Mab-3
B
C44Mab-46
C
C44Mab-3
D
C44Mab-46
E
C44Mab-3
F
C44Mab-46
G
C44Mab-3
H
C44Mab-46

Supplementary Figure S5. Immunohistochemical analysis using C_{44}Mab-3 and C_{44}Mab-46 against pancreatic adenocarcinomas and normal pancreatic tissues. After antigen retrieval, serial sections of pancreatic carcinoma tissue arrays (Catalog number: PA241c) were incubated with 1 μg/mL of C_{44}Mab-3 or C_{44}Mab-46 followed by treatment with the Envision+ kit. The color was developed using 3,3′-diaminobenzidine tetrahydrochloride (DAB), and the sections were counterstained with hematoxylin. Scale bar = 100 μm. (**A**–**F**) pancreatic adenocarcinomas; (**G, H**) normal pancreas tissues.

(**A**) C_{44}Mab-3 showed the membranous staining in pancreatic carcinoma cells with relatively larger cytoplasm, but not surrounding ductal structure of PDAC and stromal tissues. (**B**) C_{44}Mab-46 stained both type of PDAC. (**C, D**) The diffusely spread tumor cells in stroma were stained by both C_{44}Mab-3 and C_{44}Mab-46. (**E, F**) In well-differentiated PDAC, both C_{44}Mab-3 and C_{44}Mab-46 never stained the tumor cells. (**F**) In addition, stromal staining by C_{44}Mab-46 was observed in several tissues. (**G**) Importantly, normal pancreatic epithelial cells were never stained by C_{44}Mab-3.
